# Supplementary figures and images for: Comparative analysis of tardigrade locomotion across life stage, species, and disulfiram treatment
Source: PLoS One. 2024 Sep 18;19(9):e0310738. doi: 10.1371/journal.pone.0310738 (PMC11410187; doi:10.1371/journal.pone.0310738)

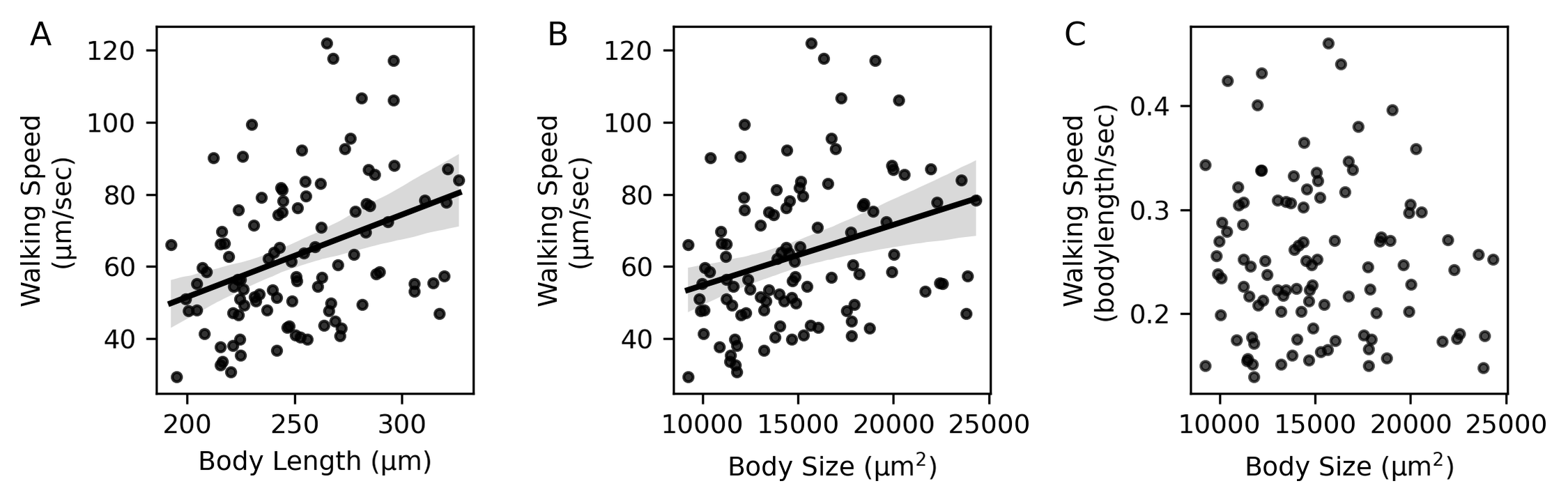

Supplement: S1 Fig — A-B. Walking speed is proportional to body length (A, Spearman’s ρ = 0.371, p<0.001, n = 103) and body area (B, Spearman’s ρ = 0.343, p<0.001, n = 103). C. When normalized to body length, walking speed is not correlated with body size. Least squares regression fit lines and 95% confidence intervals were calculated in the Python package seaborn. (TIF) [file pone.0310738.s001.tif]

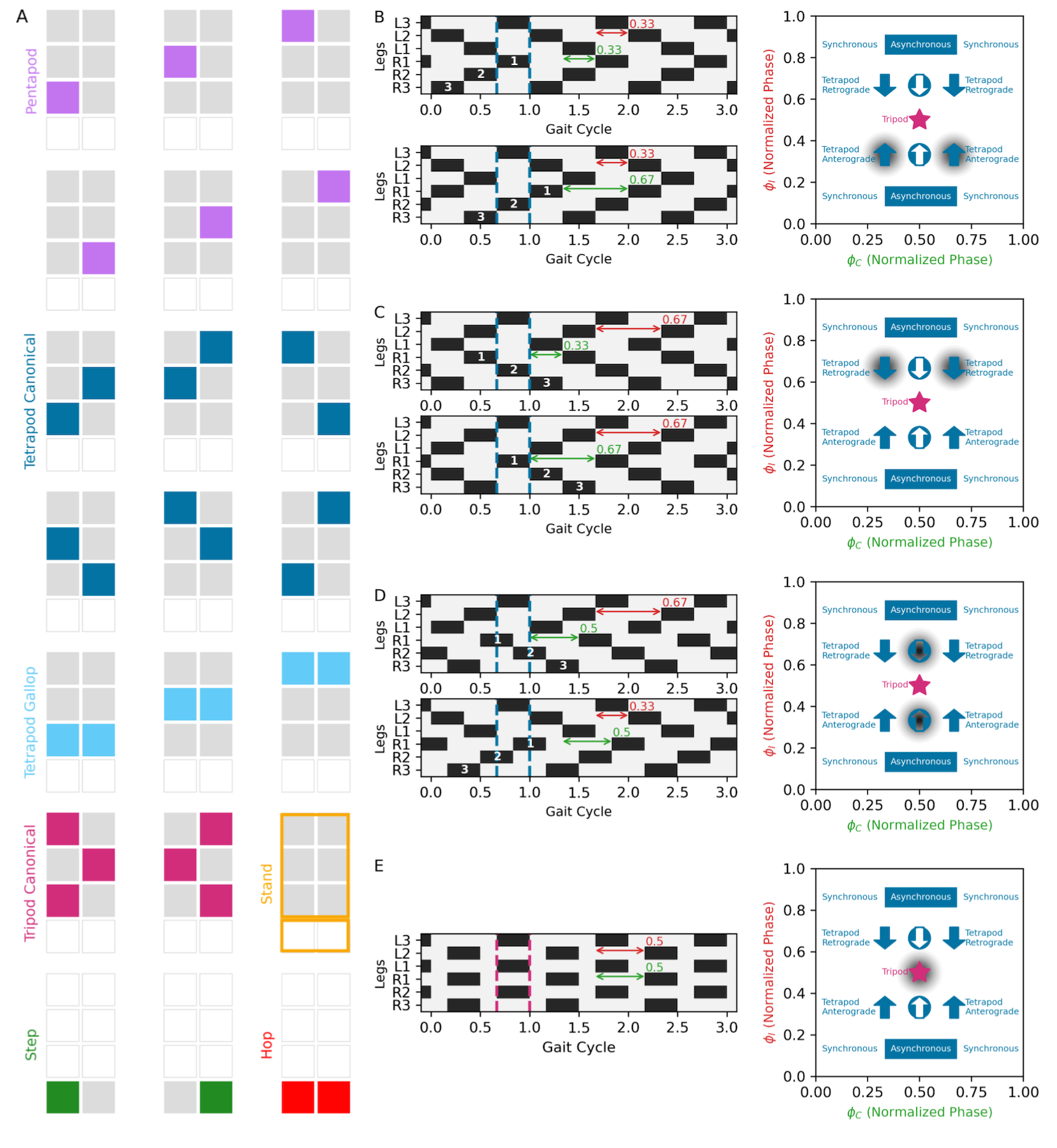

Supplement: S2 Fig — Interleg coordination patterns A. Examples of interleg coordination patterns for lateral legs and rear legs. B-E. Phase offsets determine hexapod stepping patterns. Duty factor for lateral legs is set at 2/3. B. When ϕI = 1/3 and ϕC = 1/3 or 2/3, a canonical tetrapod pattern is produced, with leg pairs initiating swing phase simultaneously (synchronous), and an anterograde (or metachronal) wave of swing initiations that travels from back to front. C. When ϕI = 2/3 and ϕC = 1/3 or 2/3, a canonical tetrapod pattern is produced, with leg pairs initiating swing phase simultaneously (synchronous), and an apparent retrograde wave of swing initiations that travels from front to back. D. When ϕI = 1/3 or 2/3 and ϕC = 1/2, a canonical tetrapod pattern is produced, with leg pairs initiating swing phase asynchronously. When ϕI = 1/3, the wave of leg swings is anterograde, while when ϕI = 2/3, the swing initiation pattern is apparently reversed. E. When ϕI = 1/2 and ϕC = 1/2, a canonical tripod gait is produced. (TIF) [file pone.0310738.s002.tif]

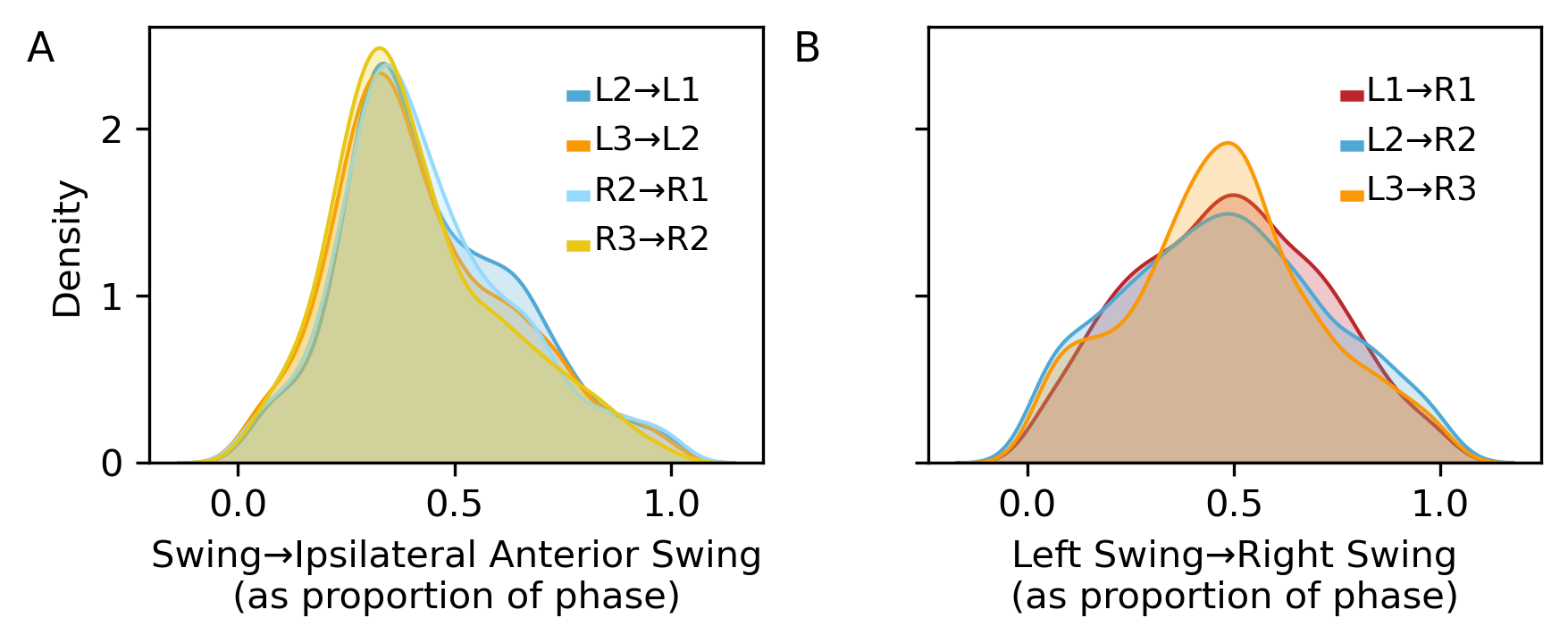

Supplement: S3 Fig — A. Distribution of ipsilateral phase offsets (ϕi) for each individual second or third pair leg. Timing of swing initiation between the indicated leg and the neighboring ipsilateral anterior leg, expressed as a fraction of the period of the posterior leg. n (strides) = 1301 for L2, 1279 for L3, 1317 for R2, 1278 for R3. B. Distribution of contralateral phase offsets (ϕC) for each left lateral leg. Timing of swing initiation between the indicated leg and the right leg within the same segment, expressed as a fraction of the period of the left leg. Probability density estimates of the distributions were calculated via the Python package seaborn. n (strides) = 1305 steps for L1, 1251 for L2, 1253 for L3. (TIF) [file pone.0310738.s003.tif]

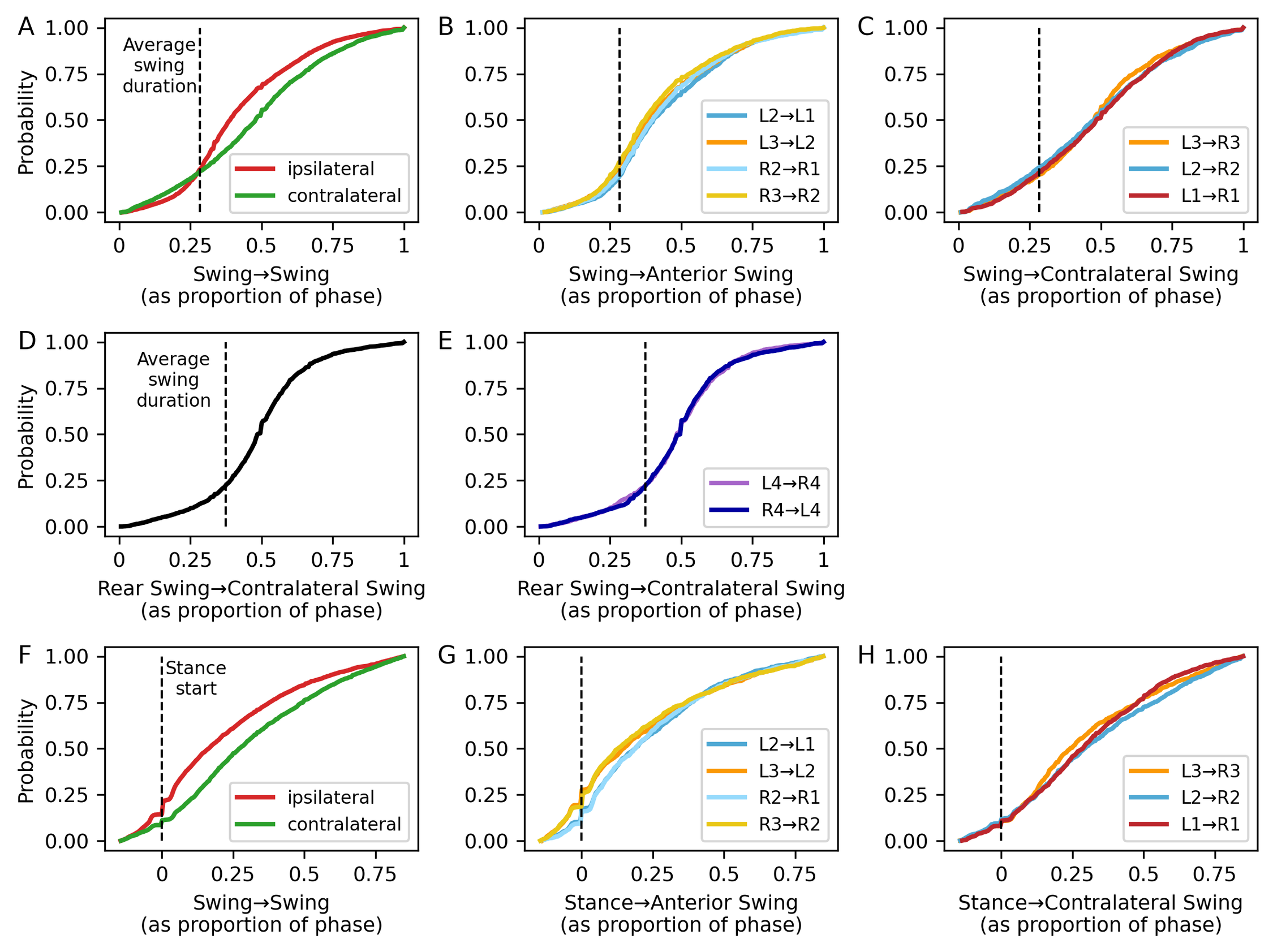

Supplement: S4 Fig — A. Cumulative distribution functions of timing of anterior ipsilateral and within-segment contralateral leg swings, normalized to the phase of the posterior or contralateral lateral leg. Average swing duration of lateral legs is shown by the dashed line, n = 5175 strides for ipsilateral phase offsets; n = 3809 strides for contralateral offsets. B. Ipsilateral swing timing for each lateral leg in the first and second leg pair, n (strides) = 1301 for L2, 1279 for R2, 1317 for L3, 1278 for R3. C. Contralateral swing timing for each left lateral leg, n (strides) = 1305 for L1, 1251 for L2, 1253 for L3. D. Cumulative distribution function of timing of contralateral leg swings for rear legs. Average swing duration of rear legs is shown by the dashed line. E. Contralateral swing timing for each rear leg, n (strides) = 1530 for L4, 1532 for R4. F. Cumulative distribution functions of timing of anterior ipsilateral and within-segment contralateral leg swings, normalized to the phase of the posterior or contralateral lateral leg. Stance onset of lateral legs is shown by the dashed line, n = 4571 strides for ipsilateral stance-swing offsets; n = 3548 strides for contralateral stance-swing offsets. G. Ipsilateral swing timing for each lateral leg in the first and second leg pair, compared to stance onset of the reference leg, n (strides) = 1145 for L2, 1154 for R2, 1145 for L3, 1127 for R3. H. Contralateral swing timing for after stance initiation of left lateral leg, n (strides) = 1203 for L1, 1184 for L2, 1161 for L3. (TIF) [file pone.0310738.s004.tif]
