# Supplementary material for: Comparative analysis of tardigrade locomotion across life stage, species, and disulfiram treatment
Source: PLoS One. 2024 Sep 18;19(9):e0310738. doi: 10.1371/journal.pone.0310738 (PMC11410187; doi:10.1371/journal.pone.0310738)
Supplement: S1 Table — (DOCX) [file pone.0310738.s005.docx]

**Supplemental Table S1. Measurements of exploratory locomotion, step kinematics, and interleg coordination.**

| **Exploratory Locomotion** | **Mean ± Std** |  | **Step kinematics** | **Mean ± Std** |  | **Interleg Coordination** | **Mean ± Std** |
| --- | --- | --- | --- | --- | --- | --- | --- |
| Original Video Length (sec) | 138.61 ± 23.45 |  | Steps Analyzed | 127.8 ± 41.49 |  | % stand (lateral legs) | 7.35 ± 5.25 |
| Body Size (µm^2^) | 15081.68 ± 3753.68 |  | Seconds Analyzed | 14.37 ± 3.21 |  | % pentapod (lateral legs) | 35.55 ± 10.66 |
| Body Length (µm) | 251.53 ± 32.25 |  | Stance duration - lateral (sec) | 0.72 ± 0.18 |  | % tetrapod canonical (lateral legs) | 37.84 ± 12.13 |
| Body Width (µm) | 79.3 ± 11.1 |  | Stance duration - rear (sec) | 0.47 ± 0.13 |  | % tetrapod gallop (lateral legs) | 6.92 ± 4.39 |
| Body Width/Length Ratio | 0.32 ± 0.02 |  | Swing duration - lateral (sec) | 0.26 ± 0.03 |  | % tetrapod other (lateral legs) | 3.98 ± 2.32 |
| Distance traveled (mm) | 5.55 ± 1.81 |  | Swing duration - rear (sec) | 0.38 ± 0.05 |  | % tetrapod total (lateral legs) | 48.74 ± 10.91 |
| Body Lengths Traveled | 22.25 ± 7.02 |  | Period - lateral (sec) | 0.99 ± 0.2 |  | % tripod canonical (lateral legs) | 2.71 ± 3.47 |
| % sustained walking | 88.68 ± 10.93 |  | Period - rear (sec) | 0.85 ± 0.15 |  | % tripod other (lateral legs) | 5.38 ± 4.04 |
| Speed (µm s^-1^) | 58.97 ± 20.7 |  | Duty factor - lateral | 0.72 ± 0.04 |  | % tripod total (lateral legs) | 8.1 ± 5.83 |
| Speed (body lengths s^-1^) | 0.23 ± 0.08 |  | Duty factor - rear | 0.54 ± 0.07 |  | % stand (rear legs) | 17.7 ± 10.73 |
| Speed (µm s^-1^ walking) | 63.28 ± 20.1 |  | Body Length / step lateral | 0.17 ± 0.02 |  | % hop (rear legs) | 6.55 ± 5.1 |
| Speed (body lengths s^-1^ walking) | 0.25 ± 0.07 |  | Body Length / step rear | 0.11 ± 0.01 |  | % step (rear legs) | 75.75 ± 8.24 |
| Bearing change (deg) s^-1^ | 16.34 ± 7.97 |  | Metachronal lag (sec) | 0.83 ± 0.26 |  | Tetrapod Coordination Strength | 0.39 ± 0.06 |
| Bearing change (deg) s^-1^ walking | 12.62 ± 4.31 |  | Metachronal lag / Period | 0.83 ± 0.14 |  | Tripod Coordination Strength | 0.17 ± 0.07 |
| Stops s^-1^ | 0.09 ± 0.08 |  | Metachronal lag Log2 Ratio | -0.01 ± 0.34 |  | Tetrapod Bout Speed (body lengths s^-1^) | 0.27 ± 0.08 |
| Turns s^-1^ | 0.05 ± 0.05 |  | Abs Metachronal lag Log2 Ratio | 0.47 ± 0.2 |  | Tripod Bout Speed (body lengths s^-1^) | 0.28 ± 0.09 |
|  |  |  | Bearing Change  ˚ s^-1^ walking | 6.56 ± 3.7 |  | Tetrapod Coordination Consistency | 0.65 ± 0.14 |
|  |  |  | ϕI (lateral legs) | 0.41 ± 0.07 |  |  |  |
|  |  |  | ϕC (lateral legs) | 0.49 ± 0.02 |  |  |  |
|  |  |  | ϕC (rear legs) | 0.5 ± 0.01 |  |  |  |
| n=103 tardigrades |  |  |  |  |  |  |  |
