# Supplementary material for: Comparative analysis of tardigrade locomotion across life stage, species, and disulfiram treatment
Source: PLoS One. 2024 Sep 18;19(9):e0310738. doi: 10.1371/journal.pone.0310738 (PMC11410187; doi:10.1371/journal.pone.0310738)
Supplement: S2 Table — (DOCX) [file pone.0310738.s006.docx]

**Supplemental Table S2. Comparison of tardigrade locomotion across life stage, species, and disulfiram treatment**

####

| **Measurement** | ***H. exemplaris* adults** | ***H. exemplaris* juveniles** | ***Ramazzottius*** | ***H. exemplaris* control** | ***H. exemplaris* disulfiram** |
| --- | --- | --- | --- | --- | --- |
| n = | 103 | 21 | 24 | 33 | 34 |
| Body Length (mm) | 0.25 ± 0.03 | 0.14 ± 0.01 | 0.22 ± 0.03 | 0.27 ± 0.03 | 0.25 ± 0.04 |
| Speed (body lengths s^-1^) | 0.23 ± 0.08 | 0.28 ± 0.1 | 0.12 ± 0.08 | 0.24 ± 0.08 | 0.13 ± 0.07 |
| Percentage of time walking | 88.68 ± 10.93 | 86.39 ± 24.21 | 39.72 ± 34.97 | 87.7 ± 16.5 | 53.21 ± 30.61 |
| Speed (mm s^-1^ walking) | 0.06 ± 0.02 | 0.04 ± 0.01 | 0.05 ± 0.02 | 0.07 ± 0.02 | 0.04 ± 0.01 |
| Speed (body lengths s^-1^ walking) | 0.25 ± 0.07 | 0.3 ± 0.08 | 0.22 ± 0.06 | 0.25 ± 0.08 | 0.18 ± 0.04 |
| Bearing change (deg) s^-1^ walking | 12.62 ± 4.31 | 13.4 ± 4.58 | 21.19 ± 5.06 | 11.96 ± 3.29 | 14.17 ± 5.29 |
| Stance duration (lateral) | 0.72 ± 0.18 | 0.67 ± 0.24 | 0.97 ± 0.2 | 0.75 ± 0.18 | 0.95 ± 0.18 |
| Swing duration (lateral) | 0.26 ± 0.03 | 0.28 ± 0.03 | 0.32 ± 0.05 | 0.26 ± 0.03 | 0.32 ± 0.04 |
| mm per step (lateral) | 0.04 ± 0.01 | 0.03 ± 0.0 | 0.05 ± 0.01 | 0.05 ± 0.01 | 0.04 ± 0.01 |
| bodylength per step (lateral) | 0.17 ± 0.02 | 0.19 ± 0.01 | 0.21 ± 0.03 | 0.17 ± 0.02 | 0.16 ± 0.01 |
| Stance duration (rear) | 0.47 ± 0.13 | 0.37 ± 0.15 | 0.67 ± 0.25 | 0.48 ± 0.11 | 0.57 ± 0.13 |
| Swing duration (rear) | 0.38 ± 0.05 | 0.34 ± 0.04 | 0.38 ± 0.06 | 0.38 ± 0.05 | 0.45 ± 0.08 |
| Abs MCL Ratio per L3 | 0.47 ± 0.2 | 0.5 ± 0.26 | 0.49 ± 0.21 | 0.46 ± 0.2 | 0.45 ± 0.2 |
| Anterior swing offsets (normalized, lateral) | 0.41 ± 0.07 | 0.46 ± 0.07 | 0.42 ± 0.07 | 0.44 ± 0.09 | 0.47 ± 0.12 |
| Opposite swing offsets (normalized, lateral) | 0.49 ± 0.02 | 0.49 ± 0.02 | 0.49 ± 0.03 | 0.48 ± 0.04 | 0.47 ± 0.05 |
| Opposite swing offsets (normalized, rear) | 0.5 ± 0.01 | 0.49 ± 0.01 | 0.47 ± 0.04 | 0.49 ± 0.01 | 0.49 ± 0.02 |
| % tetrapod canonical (lateral) | 37.84 ± 12.13 | 39.8 ± 10.5 | 28.91 ± 10.23 | 37.15 ± 11.67 | 35.39 ± 12.79 |
| Tetrapod Coordination Strength | 0.39 ± 0.06 | 0.35 ± 0.05 | 0.36 ± 0.06 | 0.38 ± 0.05 | 0.41 ± 0.07 |
| % tripod canonical (lateral) | 2.71 ± 3.47 | 6.21 ± 4.46 | 1.77 ± 1.87 | 1.33 ± 1.66 | 1.61 ± 2.21 |
| Tripod Coordination Strength | 0.16 ± 0.07 | 0.18 ± 0.06 | 0.18 ± 0.13 | 0.14 ± 0.08 | 0.16 ± 0.09 |
| % step (rear) | 75.75 ± 8.24 | 76.43 ± 5.93 | 51.08 ± 11.22 | 76.07 ± 5.61 | 76.83 ± 7.32 |
|  |  |  |  |  |  |

#### 
